# 7,8-Dihydroxyflavone Suppresses Oxidative Stress-Induced Base Modification in DNA via Induction of the Repair Enzyme 8-Oxoguanine DNA Glycosylase-1

**DOI:** 10.1155/2013/863720

**Published:** 2013-09-14

**Authors:** Ki Cheon Kim, In Kyung Lee, Kyoung Ah Kang, Ji Won Cha, Suk Ju Cho, Soo Young Na, Sungwook Chae, Hye Sun Kim, Suhkmann Kim, Jin Won Hyun

**Affiliations:** ^1^School of Medicine and Institute for Nuclear Science and Technology, Jeju National University, Jeju 690-756, Republic of Korea; ^2^Department of Obstetrics and Gynecology, Asan Medical Center, University of Ulsan College of Medicine, Seoul 138-736, Republic of Korea; ^3^Aging Research Center, Korea Institute of Oriental Medicine, Daejeon 305-811, Republic of Korea; ^4^Cancer Research Institute, Seoul National University College of Medicine, Seoul 110-799, Republic of Korea; ^5^Department of Chemistry and Chemistry Institute for Functional Materials, Pusan National University, Busan 609-735, Republic of Korea

## Abstract

The modified guanine base 8-oxoguanine (8-oxoG) is abundantly produced by oxidative stress, can contribute to carcinogenesis, and can be removed from DNA by 8-oxoguanine DNA glycosylase-1 (OGG1), which acts as an 8-oxoG glycosylase and endonuclease. This study investigated the mechanism by which 7,8-dihydroxyflavone (DHF) inhibits oxidative stress-induced 8-oxoG formation in hamster lung fibroblasts (V79-4). DHF significantly reduced the amount of 8-oxoG induced by hydrogen peroxide (H_2_O_2_) and elevated the levels of OGG1 mRNA and protein. DHF increased the binding of nuclear factor erythroid 2-related factor 2 (Nrf2) to antioxidant response element sequences in the upstream promoter region of OGG1. Moreover, DHF increased the nuclear levels of Nrf2, small Maf proteins, and the Nrf2/small Maf complex, all of which are decreased by H_2_O_2_ treatment. Likewise, the level of phosphorylated Akt, which activates Nrf2, was decreased by H_2_O_2_ treatment but restored by DHF treatment. The levels of OGG1 and nuclear translocation of Nrf2 protein were decreased upon treatment with PI3K inhibitor or Akt inhibitor, and DHF treatment did not restore OGG1 and nuclear Nrf2 levels in these inhibitor-treated cells. Furthermore, PI3K and Akt inhibitors abolished the protective effects of DHF in cells undergoing oxidative stress. These data indicate that DHF induces OGG1 expression via the PI3K-Akt pathway and protects cells against oxidative DNA base damage by activating DNA repair systems.

## 1. Introduction

8-Oxoguanine (8-OxoG) is a major form of DNA damage produced by oxidative stress [1]. This modification can induce DNA mutations or alterations, which can eventually lead to diseases including cancer [2–8]. Under normal physiological conditions in all aerobic organisms, a balance is maintained between endogenous oxidants and numerous enzymatic and nonenzymatic antioxidant defenses [9]. Single-base oxidations, such as 8-oxoG, induced by extreme oxidative conditions are repaired by the base excision repair (BER) system [10], which recognizes and removes damaged and irrelevant bases [11].

8-Oxoguanine DNA glycosylase-1 (OGG1), a BER enzyme, repairs 8-oxoG by cleaving the glycosidic bond of the 8-oxoG lesion and causing a strand break in the DNA backbone [12, 13]. The OGG1 promoter region contains antioxidant response elements (ARE) that are recognized by nuclear factor erythroid 2-related factor 2 (Nrf2), a transcription factor that is essential for ARE-mediated induction of genes encoding phase II detoxification and oxidative stress-response enzymes [14–16]. Prior to activation, Nrf2 resides in the cytosol. To function as a transcription factor, Nrf2 must first dissociate from Keap1, which degrades Nrf2 by targeting it for ubiquitination and subsequently translocate to the nucleus [17]. The primary signal that causes Nrf2 to dissociate from Keap1 is phosphorylation, mediated by the phosphoinositide 3-kinase (PI3K)/Akt pathway [18, 19]. Once in the nucleus, Nrf2 must form a heterodimer with a small Maf protein in order to bind the ARE with high affinity and activate gene expression via this response element [17]. 

The polyphenolic compound 7,8-dihydroxyflavone (DHF) is a member of a diverse class of secondary plant metabolites [20]. We recently demonstrated that DHF prevents oxidative stress-induced genotoxicity by scavenging reactive oxygen species (ROS) and enhancing Akt activity [21]. However, very few reports have addressed the mechanisms underlying the influence of DHF on DNA repair of oxidative lesions in the DNA. In this study, we focused on the cytoprotective effect of DHF against H_2_O_2_-induced DNA base damage and its effects on the activity of the repair enzyme OGG1.

## 2. Materials and Methods

### 2.1. Reagents

7,8-Dihydroxyflavone (DHF) was purchased from Tokyo Chemical Industry Co. (Chuo-ku, Tokyo, Japan). The plasmid containing the OGG1-promoter luciferase construct was a kind gift from Professor Ho Jin You (Chosun University, Gwangju, Republic of Korea). Avidin-conjugated tetramethylrhodamine isothiocyanate (TRITC), [3-(4,5-dimethylthiazol-2-yl)-2,5-diphenyltetrazolium] bromide (MTT) and an antibody against OGG1 were purchased from Sigma-Aldrich Corporation (St. Louis, MO, USA). Antibodies against Nrf2, small Maf, phospho-Akt, Akt, TATA box-binding protein (TBP), and *β*-actin were purchased from Santa Cruz Biotechnology (Santa Cruz, CA, USA). LY294002 and Akt inhibitor IV were purchased from Calbiochem (San Diego, CA, USA). 

### 2.2. Cell Culture

Lung is frequently contacted to oxygen and ROS [22, 23]. Chinese hamster lung fibroblasts (V79-4) were used to elucidate the protective effects of DHF against the oxidative stress- induced DNA base modification. V79-4 cells were obtained from the American Type Culture Collection. Cells were maintained at 37°C in an incubator in a humidified atmosphere of 95% air/5% CO_2_. Culture medium was Dulbecco's modified Eagle's medium containing 10% heat-inactivated fetal calf serum, streptomycin (100 *μ*g/mL), and penicillin (100 units/mL).

### 2.3. Measurement of the 8-OxoG Level

We previously reported that 40 *μ*M DHF displayed substantial intracellular ROS scavenging activity and was nontoxic to V79-4 cells [21] and that treatment of cells with 1 mM H_2_O_2_ resulted in 50–60% viability [21, 24]. These concentrations of DHF and H_2_O_2_ were considered optimum for this present study. 

Cells were treated with 40 *μ*M DHF for 1 h, and then 1 mM H_2_O_2_ was added to the medium. After 24 h, cellular DNA was isolated using the DNAzol reagent (Life Technologies, Grand Island, NY, USA) and quantified using a spectrophotometer. The level of 8-hydroxy-2-deoxyguanosine (8-ohdG; a nucleoside of 8-oxoG) in DNA, considered to represent the 8-oxoG level, was determined using the Bioxytech 8-OHdG ELISA kit, in which 8-ohdG is coated on well surface, from OXIS Health Products (Portland, OR, USA). The 8-oxoG level was also estimated using a fluorescence-based binding assay [25]. Cells were fixed and permeabilized with ice-cold methanol for 15 min and then incubated with avidin-conjugated TRITC (fluorescent dye) for 1 h at room temperature; avidin binds 8-oxoG and 8-ohdG with high affinity. The resultant TRITC signal (representing the 8-oxoG level) was visualized using a fluorescence microscope. 

### 2.4. Transient Transfection and OGG1-Promoter Luciferase Assay

The nucleotide sequence region for OGG1 promoter in the plasmid containing the OGG1-promoter luciferase construct is from −1947 to +126, which contains Nrf2 binding site (from −5 to +13) [14]. Cells were transiently transfected with a luciferase-reporter plasmid under the control of the OGG1 promoter, using the transfection reagent DOTAP (Roche, Mannheim, Germany). After overnight transfection, cells were treated with 40 *μ*M DHF for 1 h, and then 1 mM H_2_O_2_ was added to the medium. After 24 h, cells were lysed with reporter lysis buffer (Promega, Madison, WI, USA). The lysate supernatant was mixed with the luciferase assay reagent, and the mixture was placed in a luminometer to measure the light produced.

### 2.5. Reverse Transcriptase-Polymerase Chain Reaction (RT-PCR)

Total RNA was isolated from cells using easy-BLUE (iNtRON Biotechnology, Kyounggi, Republic of Korea). PCR conditions were as follows: 94°C for 2 min, 35 cycles of 94°C for 20 sec, 58°C for 30 sec, and 72°C for 1 min; and 72°C for 5 min. The primer pairs (Bioneer Corporation, Daejeon, Republic of Korea) were as follows: mouse OGG1, sense 5′-GCAGAGCCCTGCTCACTGGA-3′ and antisense 5′-CGAGGATGGCTTTGGCACTG-3′; mouse *β*-actin, sense 5′-GTGGGCCGCCCTAGGCACCAGG-3′ and antisense 5′-GGAGGAAGAGGATGCGGCAGTG-3′. Amplified products were resolved by 1% agarose gel electrophoresis, stained with ethidium bromide, and photographed under ultraviolet light.

### 2.6. Extraction of Total Cellular Proteins and a Nucleus Protein Fraction

5 × 10^5^ cells were seeded into 60 mm dishes containing a total of 5 mL of medium. To extract total cellular proteins, cells were lysed on ice for 30 min in 150 *μ*L of PRO-PREP (iNtRON Biotechnology) and centrifuged at 13,000 rpm for 30 min. To extract nuclear proteins, cells were lysed using the Subcellular Protein Fractionation Kit (Thermo Scientific, Milwaukee, WI, USA). 

### 2.7. Quantitative Real-Time PCR

cDNA synthesis was performed with 2 *μ*g RNA using a Superscript kit (Invitrogen, Carlsbad, CA, USA). Quantitative real-time PCR was performed with 2 × SYBR Green Mastermix (Invitrogen, Carlsbad, CA, USA) and 900 nM primers for OGG1 and 18s rRNA. The thermal cycling parameters were as follows: an initial cycle of Taq activation for 1 min at 94°C, 1 min at 55°C, and 1 min at 72°C and optimized annealing temperature 5 sec at 58–60°C, 30 sec at 72°C, and a detection step of 8 sec at 80°C. Reactions were performed in duplicate, and the specificity was monitored using melting curve analysis after cycling. The following primers were designed by Bioneer Co. Ltd. (Seoul, Republic of Korea) (5′–3′) 18s rRNA, 5′-CAGCCACCCCAGATTGAGCA-3′ and 5′-TAGTAGCGACGGGCGGTGTG-3′; (5′–3′) OGG1, 5′-TACCGAGGAGACAAGAGCCA-3′ and 5′-GGCTATACAGCTGAGCCAGG-3′. All data were tested first for normality, and data with a nonnormal distribution were subjected to square root transformation prior to statistical analyses. The comparative cycle threshold (Ct) method was used to calculate the relative changes in gene expression in the iQ5 real-time PCR system (Bio-Rad Laboratories, Hercules, CA, USA). The 2-delta-delta Ct value was calculated after 18s rRNA normalization [26].

### 2.8. Western Blotting

Cell or nuclear lysates were collected, and protein concentrations were determined using the Bradford reagent. Aliquots of the lysates (40 *μ*g of protein) were boiled for 5 min and electrophoresed on 10% SDS-polyacrylamide gels. Gels were transferred onto nitrocellulose membranes (Bio-Rad, CA, USA). Membranes were then incubated with the indicated primary antibodies and further incubated with secondary immunoglobulin G-horseradish peroxidase conjugates. Protein bands were visualized by developing the blots using an Enhanced Chemiluminescence Western Blotting Detection Kit (Amersham, Buckinghamshire, UK) and exposing the membranes to X-ray film.

### 2.9. Immunoprecipitation

Cell lysates were mixed with the anti-Nrf2 antibody and shaken at 4°C overnight. Next, 30 *μ*L of protein G-agarose beads was added to the lysates, and the mixture was shaken for 2 h at 4°C. The lysates were centrifuged at 6000 rpm for 5 min, and the protein G-agarose beads were collected. Collected beads were incubated with elution buffer for 30 min on ice and then centrifuged at 13,000 rpm for 5 min. Supernatants were collected and protein concentrations were determined using the Bradford reagent.

### 2.10. Immunocytochemistry

Cells plated on coverslips were fixed with 1% paraformaldehyde for 30 min and permeabilized with 2% Triton X-100 in PBS for 2.5 min. Cells were treated with blocking medium (1% bovine serum albumin in PBS) for 1 h and incubated with Nrf2 antibody diluted in blocking medium for 2 h. Immunoreactive primary Nrf2 antibody was detected by a 1 : 200 dilution of FITC-conjugated secondary antibody (Jackson ImmunoResearch Laboratories, West Grove, PA, USA) for 1 h. After washing with PBS, stained cells were mounted onto microscope slides in mounting medium with DAPI (Vector, Burlingame, CA, USA). Images were collected using the LSM 510 program on a Zeiss confocal microscope.

### 2.11. Cell Viability

Cells were seeded in a 24-well plate at a density of 3.5 × 10^4^ cells/well and pretreated with 50 *μ*M LY294002 or 1 *μ*M Akt IV for 1 h, followed by treatment with 40 *μ*M DHF for 1 h and treatment with 1 mM H_2_O_2_ for an additional 24 h. MTT (150 *μ*L of a 2 mg/mL stock solution) was then added to each well to yield a total reaction volume of 500 *μ*L. After incubating for 4 h, the plate was centrifuged at 1500 rpm for 5 min, and the supernatants were aspirated. The formazan crystals in each well were dissolved in 1 mL dimethylsulfoxide, and the resultant solutions were transferred to 96-well spectrometry plates, and the *A*_540_ was read on a scanning multiwell spectrophotometer [27]. 

### 2.12. Statistical Analysis

All measurements were made in three independent experiments, and all values are represented as the mean ± standard error. The results were subjected to an analysis of variance using Tukey's test for analysis of differences. Statistical significance was set at *P* < 0.05.

## 3. Results

### 3.1. DHF Suppresses Formation of 8-OxoG by Oxidative Stress

**Figure 1:**
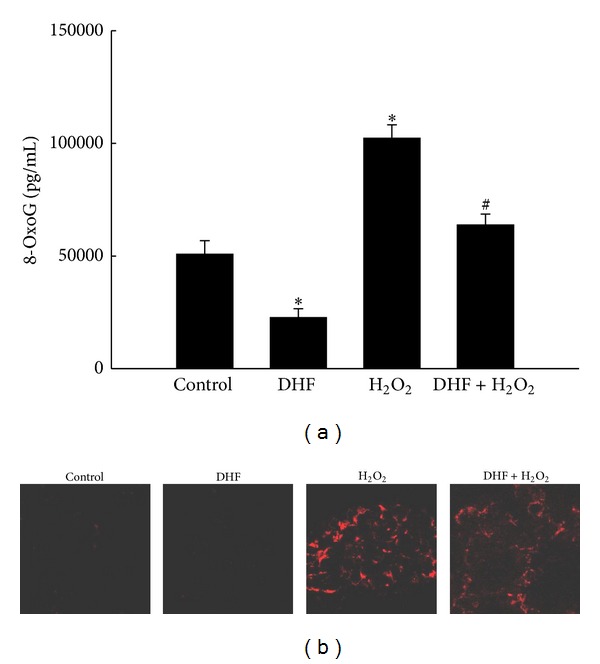
DHF reduces H_2_O_2_-induced 8-oxoG formation. (a) Cells were treated with 40 *μ*M DHF for 1 h and then incubated with 1 mM H_2_O_2_ for an additional 24 h. *Significantly different from control cells (*P* < 0.05); ^#^significantly different from H_2_O_2_-treated cells (*P* < 0.05). (b) Binding of avidin-TRITC, which reflects 8-oxoG levels, was visualized with a fluorescence microscope.

8-OxoG is one of the major forms of ROS-induced oxidative base lesions in DNA and has therefore been widely used as a biomarker for oxidative stress and carcinogenesis [28]. We measured the 8-oxoG level in DNA by ELISA using specific antibodies against 8-oxoG. The 8-oxoG level was significantly higher in H_2_O_2_-treated cells than in control cells, but DHF pretreatment prior to H_2_O_2_ treatment significantly suppressed 8-oxoG formation (Figure 1(a)). We also estimated the amount of 8-oxoG using a fluorescence-based binding assay with an avidin-conjugated TRITC reagent [25]. The fluorescence intensity was higher in H_2_O_2_-treated cells than in control cells, and fluorescence was reduced in H_2_O_2_-treated cells that had been pretreated with DHF (Figure 1(b)). These results indicate that DHF decreased the 8-oxoG level in H_2_O_2_-treated cells.

### 3.2. DHF Increases OGG1-Promoter Transcriptional Activity and Restores OGG1 mRNA and Protein Expression That Are Reduced by H_2_O_2_ Treatment

**Figure 2:**
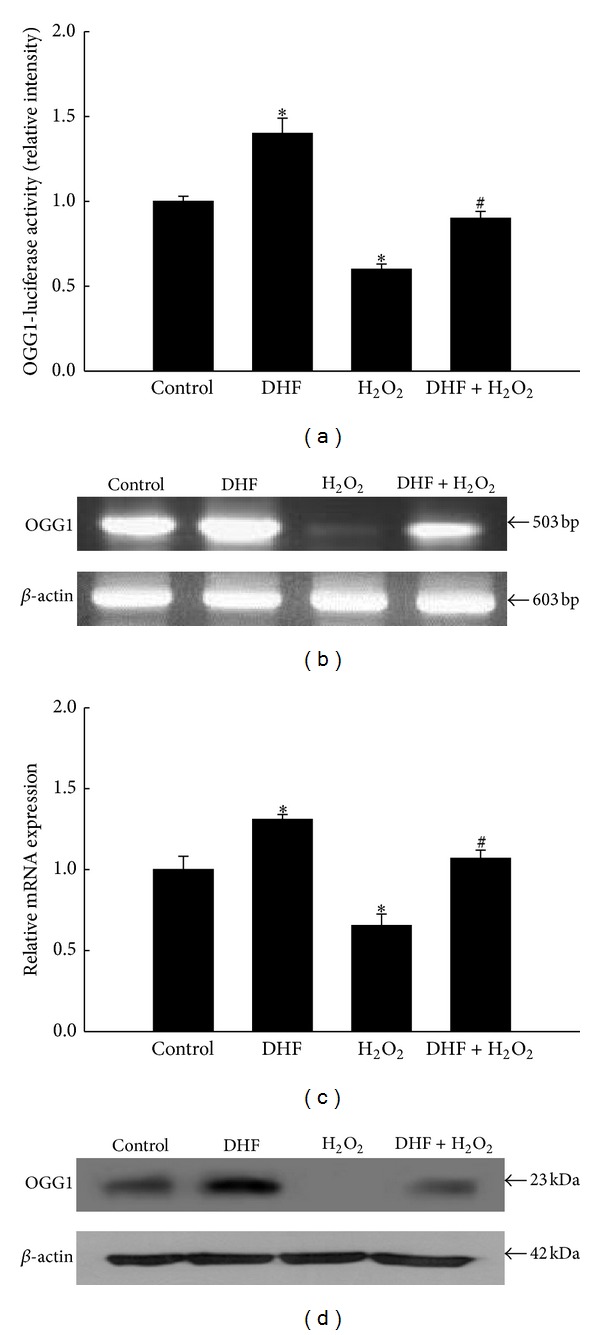
DHF induces OGG1 promoter activity and OGG1 protein expression. (a) After overnight transfection with the OGG1-promoter luciferase vector, cells were treated with 40 *μ*M DHF for 1 h, incubated with 1 mM H_2_O_2_ for an additional 24 h, and then subjected to luciferase assays. *Significantly different from control cells (*P* < 0.05); ^#^significantly different from H_2_O_2_-treated cells (*P* < 0.05). (b) Cells were treated with 40 *μ*M DHF for 1 h and then incubated with 1 mM H_2_O_2_ for an additional 24 h. OGG1 mRNA levels were detected by RT-PCR analysis and (c) quantitative real-time PCR. *Significantly different from control cells (*P* < 0.05); ^#^significantly different from H_2_O_2_-treated cells (*P* < 0.05). (d) OGG1 protein levels were detected by western blot analysis.

Excessive oxidative stress can overwhelm cellular repair systems. OGG1, an enzyme that repairs oxidized DNA bases, is the primary enzyme responsible for the excision of 8-oxoG lesions induced by oxidative stress [29]. The transcriptional activity of the OGG1 promoter was strongly suppressed in H_2_O_2_-treated cells (Figure 2(a)); however, this activity was restored by pretreatment with DHF. The level of OGG1 mRNA, determined by RT-PCR and quantitative real-time PCR, was lower in H_2_O_2_-treated cells than in control cells; however, DHF pretreatment partially restored the OGG1 mRNA level (Figures 2(b) and 2(c)). Furthermore, the level of OGG1 protein was higher in H_2_O_2_-treated cells pretreated with DHF than in cells treated with H_2_O_2_ alone (Figure 2(d)). These data demonstrate that DHF can reverse the OGG1 transcription and OGG1 protein expression reduced by H_2_O_2_ treatment.

### 3.3. DHF Reverses Nuclear Levels of Nrf2, Small Maf-, and the Nrf2-Small Maf Complex Reduced by H_2_O_2_ Treatment

**Figure 3:**
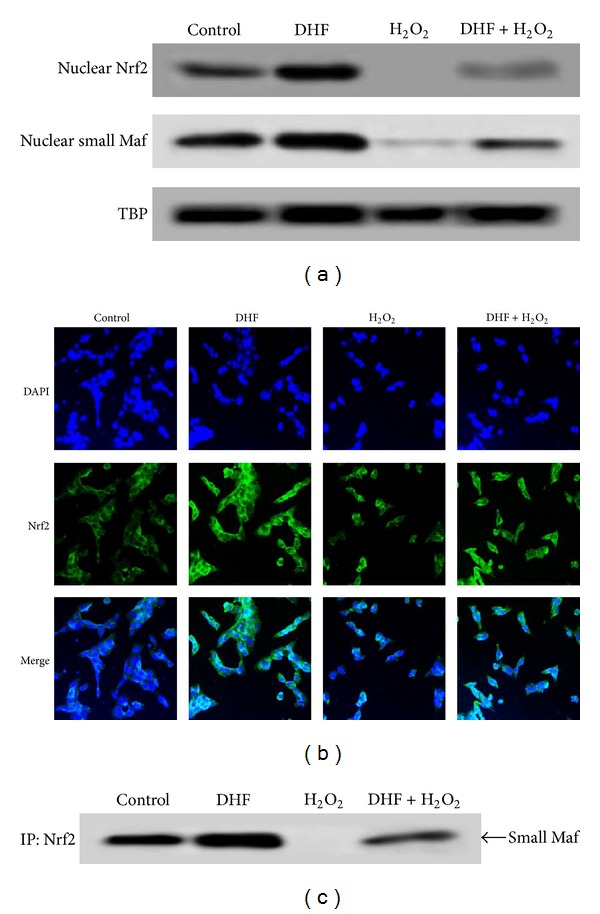
DHF induces binding of Nrf2 to small Maf transcription factor. (a) Cells were treated with 40 *μ*M DHF for 1 h and then incubated with 1 mM H_2_O_2_ for an additional 12 h. Nuclear extracts were electrophoresed, and nuclear Nrf2, small Maf protein, and TBP were detected using specific antibodies. (b) Confocal images of Nrf2 antibody and FITC-conjugated secondary antibody staining indicate the location of Nrf2 protein (green); DAPI staining indicates the nucleus (blue). The merged images reveal the nuclear or cytosol location of Nrf2 protein. (c) Nuclear extracts were immune-precipitated with an anti-Nrf2 antibody and subjected to western blotting using an antibody against small Maf protein.

The *OGG1* promoter region contains specific transcription factor binding sites (ARE sequences) for Nrf2 [14, 30]. Nrf2 binds to the ARE with high affinity only as a heterodimer with a small Maf protein, and the Nrf2/small Maf complex activates gene expression directly through the ARE [17]. To analyze the levels of Nrf2 and small Maf protein in the nucleus, the expression of nuclear proteins were measured by western blotting. H_2_O_2_ treatment decreased the levels of Nrf2 and small Maf proteins in the nucleus; however, DHF treatment restored these proteins to control levels (Figure 3(a)). And H_2_O_2_ treatment decreased the Nrf2 protein translocation from cytoplasm to nucleus; however, DHF treatment restored it (Figure 3(b)). To determine whether Nrf2 was bound to small Maf protein in the nucleus, nuclear lysates were immune-precipitated with an anti-Nrf2 antibody and subjected to western blotting with an antibody against small Maf protein. H_2_O_2_ treatment decreased the binding of Nrf2 to small Maf protein, and DHF treatment restored this binding (Figure 3(c)). These results indicate that DHF treatment can increase OGG1 expression by increasing the levels of Nrf2, small Maf, and Nrf2/small Maf complex in the nucleus. 

### 3.4. The Induction of OGG1 by DHF Is Mediated via the PI3K/Akt Pathway

**Figure 4:**
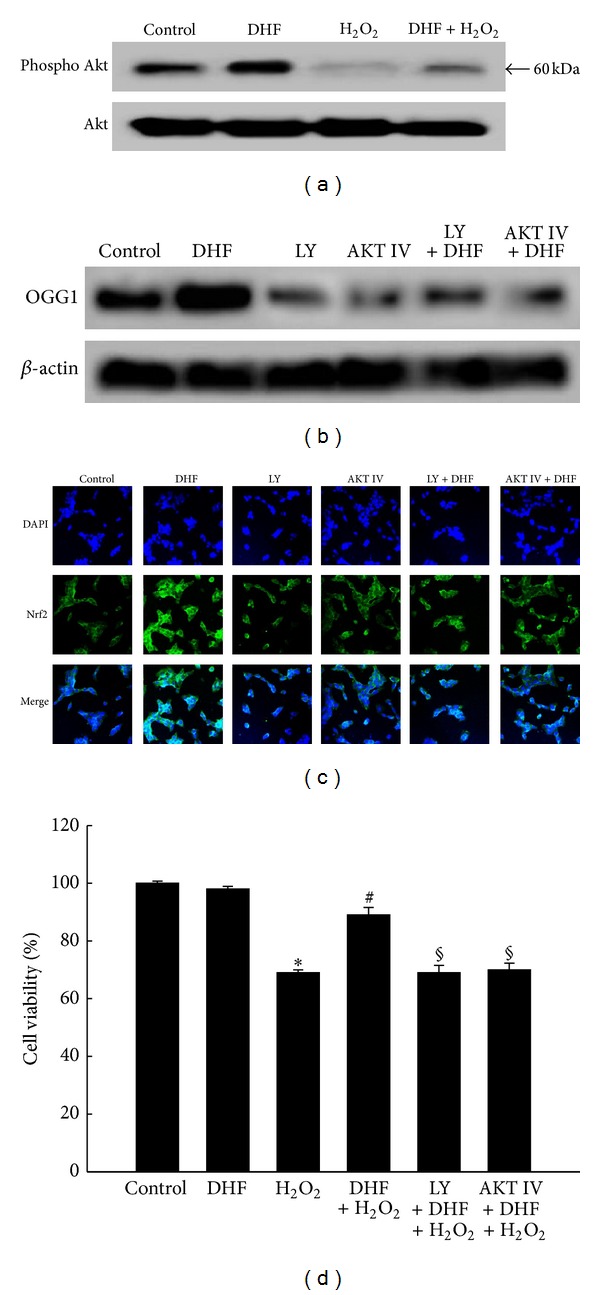
DHF induces OGG1 expression via the PI3K/Akt signaling pathway and improves cell survival. (a) Cells were treated with 40 *μ*M DHF for 1 h and then incubated with 1 mM H_2_O_2_ for an additional 12 h. Cell lysates were electrophoresed, and Akt and phospho-Akt were detected using specific antibodies. (b) Cells were pretreated with 50 *μ*M LY294002 or 1 *μ*M Akt inhibitor IV for 1 h and then treated with 40 *μ*M DHF for an additional 24 h. OGG1 was detected by western blotting. (c) Confocal images of Nrf2 antibody and FITC-conjugated secondary antibody staining indicate the location of Nrf2 protein (green); DAPI staining indicates the nucleus (blue). The merged images of DHF treatment in PI3K or Akt inhibitor-treated cells reveal the cytosolic location of Nrf2 protein. (d) Cells were pretreated with 50 *μ*M LY294002 or 1 *μ*M Akt inhibitor IV for 1 h, treated with 40 *μ*M DHF for 1 h, and then incubated with 1 mM H_2_O_2_ for an additional 24 h. Cell viability was assessed by the MTT assay. *Significantly different from control cells (*P* < 0.05); ^#^significantly different from H_2_O_2_-treated cells (*P* < 0.05); ^§^significantly different from H_2_O_2_-treated cells pretreated with DHF (*P* < 0.05).

The PI3K/Akt pathway is a major signaling process critical for cell survival during oxidative stress. The OGG1 promoter region contains Nrf2-binding sites, and the PI3K/Akt pathway is involved in the upregulation of OGG1 [15]. The level of phospho-Akt was decreased upon H_2_O_2_ treatment; however, DHF treatment reversed the reduction in phospho-Akt (Figure 4(a)). Treatment with LY294002 (a PI3K inhibitor) or Akt inhibitor IV attenuated the ability of DHF treatment to induce OGG1 expression (Figure 4(b)). Both PI3K and Akt inhibitors suppressed the translocation of Nrf2 to the nucleus that was induced by treatment with DHF (Figure 4(c)). Likewise, cell viability was inhibited by H_2_O_2_ treatment, and DHF treatment restored viability, but the restoration of cell viability by DHF was blocked by treatment with PI3K or Akt inhibitor (Figure 4(d)). These results indicate that the PI3K/Akt pathway partially regulates Nrf2 transcriptional activity and OGG1 expression, thereby affecting cell viability. 

## 4. Discussion

In a previous study, we demonstrated that DHF exerts cytoprotective properties against oxidative stress-induced genotoxic events, such as DNA base modification and DNA breaks, by scavenging intracellular ROS and enhancing Akt activity [21]. 8-OxoG lesions induced by oxidative stress can be repaired by OGG1 enzyme under the normal cellular redox state. During DNA synthesis in an abnormal redox state induced by large perturbations, 8-oxoG forms a mismatched base pair with adenine, giving rise to a G : C to T : A transversion mutation, potentially altering gene function and resulting in cell death [3, 4, 31]. OGG1 is a major glycosylase involved in BER of oxidative DNA damage. Cells transfected with siRNA to OGG1 exhibited the reduced OGG1 activity, which was accompanied by delayed repair of xanthine oxidase-induced mitochondrial DNA damage and increased apoptosis [32]. And Liu et al. (2011) demonstrated that cortical neurons isolated from OGG1-deficient mice were more vulnerable to oxidative stress than that of normal OGG1 mice, and OGG1-deficient mice developed cortical infarcts and behavior deficits after cerebral artery occlusion [33]. 

The OGG1 promoter contains ARE sequences, that is, Nrf2-binding sequences, which are activated under antioxidant or electrophilic conditions in a normal redox state [14, 15]. Nrf2 translocates into the nucleus and heterodimerizes with small Maf, which are also basic leucine-zipper transcription factor; the resultant Nrf2/small Maf complex acts as a transcription factor for ARE-driven genes [34]. 

Under nonstressed conditions, Nrf2 is anchored in the cytoplasm by binding to Keap1, which facilitates the ubiquitination and subsequent proteolysis of Nrf2 [16, 35]. Upon exposure to various stresses, Nrf2 is released from Keap1 and translocates into the nucleus [36, 37]. Nrf2-Keap1 dissociation and Nrf2 signaling are initiated by phosphorylation of Nrf2 at serine 40 and at specific threonine residues, as well as by the modification of cysteine residues in Keap1 [17, 38]. One of the signals that trigger Nrf2-Keap1 dissociation and phosphorylation of Nrf2 is mediated by various kinases, including mitogen-activated protein kinases, protein kinase C, PI3K/Akt, casein kinase-2, and PKR-like endoplasmic reticulum kinase [39]. 

The mild ROS conditions stimulate cellular antioxidant system which protects against oxidative stress in general [9], but extreme ROS can destroy the cytoprotective defense mechanism by emasculation of antioxidant and/or DNA repair systems [40]. In the present study, H_2_O_2_ (1 mM) inhibited OGG1 expression which eliminates the oxidized guanine. This phenomenon supposes that extreme ROS can destroy the cytoprotective defense mechanism while pretreatment of DHF may offer to enhance the antioxidant and/or DNA repair systems. Also, a proper dose of H_2_O_2_ can induce the Nrf2-translocalization to the nucleus [41], but extreme concentrations of H_2_O_2_ may inhibit it. Our data demonstrated that H_2_O_2_ treatment reduced the level of Nrf2, small Maf, and Nrf2/small Maf in the nucleus, but DHF pretreatment restored these levels. It is estimated that DHF pretreatment can transfer Nrf2 to the nucleus and activate the Nrf2 transcriptional activity in this study. In H_2_O_2_-treated cells, DHF pretreatment restored Akt phosphorylation and OGG1 expression, but this effect was diminished by inhibition of the PI3K/Akt pathway, suggesting that restoration of OGG1 expression by DHF treatment is partially regulated by a PI3K/Akt-dependent pathway. 

**Figure 5:**
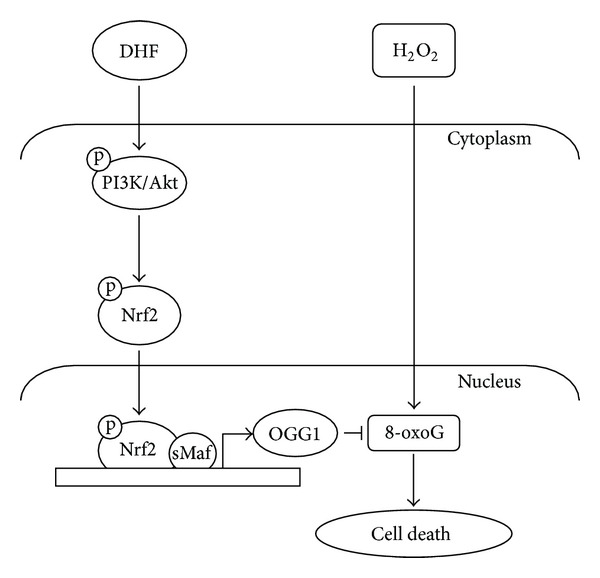
The proposed model of OGG1 induction by DHF treatment and its cytoprotective effect against cell death. DHF activates the OGG1 via the PI3K/Akt/Nrf2 signaling pathway and removes 8-oxoG in response to oxidative stress, resulting in prevention against cell death induced by oxidative damage.

Previous study suggested that PI3K/Akt upregulates phase II enzyme NAD(P)H:quinone oxidoreductase and HO-1 via phosphorylation of Nrf2 transcription factors [42, 43]. Also, it has reported that upstream kinases including PI3K/Akt phosphorylate specific serine or threonine residues present in Nrf2, resulting in facilitating the nuclear localization of Nrf2 [44]. Furthermore, hyperoxia induced ROS-EGFR-PI3K-Akt signaling cascades, which phosphorylates an Nrf2 in pulmonary epithelial cells [45]. PI3K/Akt, which plays a key role in multiple cellular processes, regulates cellular survival and metabolism by binding and regulating many downstream effectors. PI3K/Akt signaling was involved in ROS-mediated self-renewal and neurogenesis in neural stem cells [46] and in proliferation of human coronary artery endothelial cells [47]. Therefore, OGG1 induction by DHF treatment partially may be involved via PI3K/Akt/Nrf2 pathway. Furthermore, the cytoprotective effects of DHF during H_2_O_2_ treatment were also attenuated by PI3K/Akt inhibition. Taken together, the data presented here demonstrate that DHF activates the BER system via the PI3K/Akt/Nrf2 signaling pathway in response to oxidative stress and thereby prevents cell death induced by oxidative damage (Figure 5).
